# Assessing the effect of wind farms in fauna with a mathematical model

**DOI:** 10.1038/s41598-020-71758-5

**Published:** 2020-09-08

**Authors:** Pablo Refoyo Román, Cristina Olmedo Salinas, Benito Muñoz Araújo

**Affiliations:** Biodiversity, Ecology & Evolution Department, Biological Sciences Faculty, Complutense University of Madrid, José Antonio Novais, 12, 28040 Madrid, Spain

**Keywords:** Zoology, Ecology, Environmental sciences

## Abstract

Energy production by wind turbines has many advantages. The wind is a renewable energy that does not emit greenhouse gases and has caused a considerable increase in wind farms around the world. However, this type of energy is not completely free of impact. In particular, wind turbines displace and kill a wide variety of wild species what forces us to plan their location well. In any case, the determination of the effects of wind farms on fauna, especially the flying one, is difficult to determine and depends on several factors. In this work, we will try to establish a mathematical algorithm that allows us to combine all variables that affect the species with the idea of quantifying the effect that can cause the installation of a wind farm with certain characteristics in a given place. We have considered specific parameters of wind farms, the most relevant environmental characteristics related to the location of the wind farm, and morphological, ethological and legal characteristics in the species. Two types of assessment are established for the definitive valuation. Total Assessment and Weighted Assessment. Total Valuation is established based on a reference scale that will allow us to establish categories of affection for the different species while Weighted valuation allows us to establish which species are most affected.

## Introduction

Energy production by wind turbines has many advantages. The wind is a renewable energy that does not emit greenhouse gases, which has allowed it to receive aid from national governments and international institutions, and has caused a considerable increase in wind farms around the world^1^. The growth of wind energy has been enormous in Europe. For example, a wind capacity of 15,638 MW installed during 2017 represents an increase of 25% compared to 2016^2^. The country that has most contributed to this increase is Germany with 6.6 GW.

Certainly, the extension of this kind of energy is extremely beneficial to combat modern environmental problems, especially climate change. However, this type of energy is not completely free of impact. In particular, wind turbines displace and kill a wide variety of wild species what forces them to plan well their location^3^. These impacts happen in all phases of the process, both in the construction of the facilities and associated power lines as well as in the phases of exploitation and dismantling^4^. In the United States, for example, it is estimated that wind energy causes 0.01% of avian mortality due to anthropogenic causes and also up to 300,000 individuals can be affected in 2030^5^.

In any case, the determination of the effects of wind farms on fauna, especially the flying one, is difficult to determine and depends on several factors^6–12^. There are some studies comprising wide periods of time that suggest that the problems of the wind farms on the flying fauna highly depends on the suitability of their location and the environmental conditions^8^. Equally, the Council of Europe in its recommendations to minimize the adverse effects of wind farms on birds and bats (Recommendation on minimizing adverse effects of wind power generation on birds and bats, Council of Europe 2004) highlights the appropriate choice of the location of each wind farm as a critical criterion to avoid the harmful effects on the avifauna.

**Figure 1:**
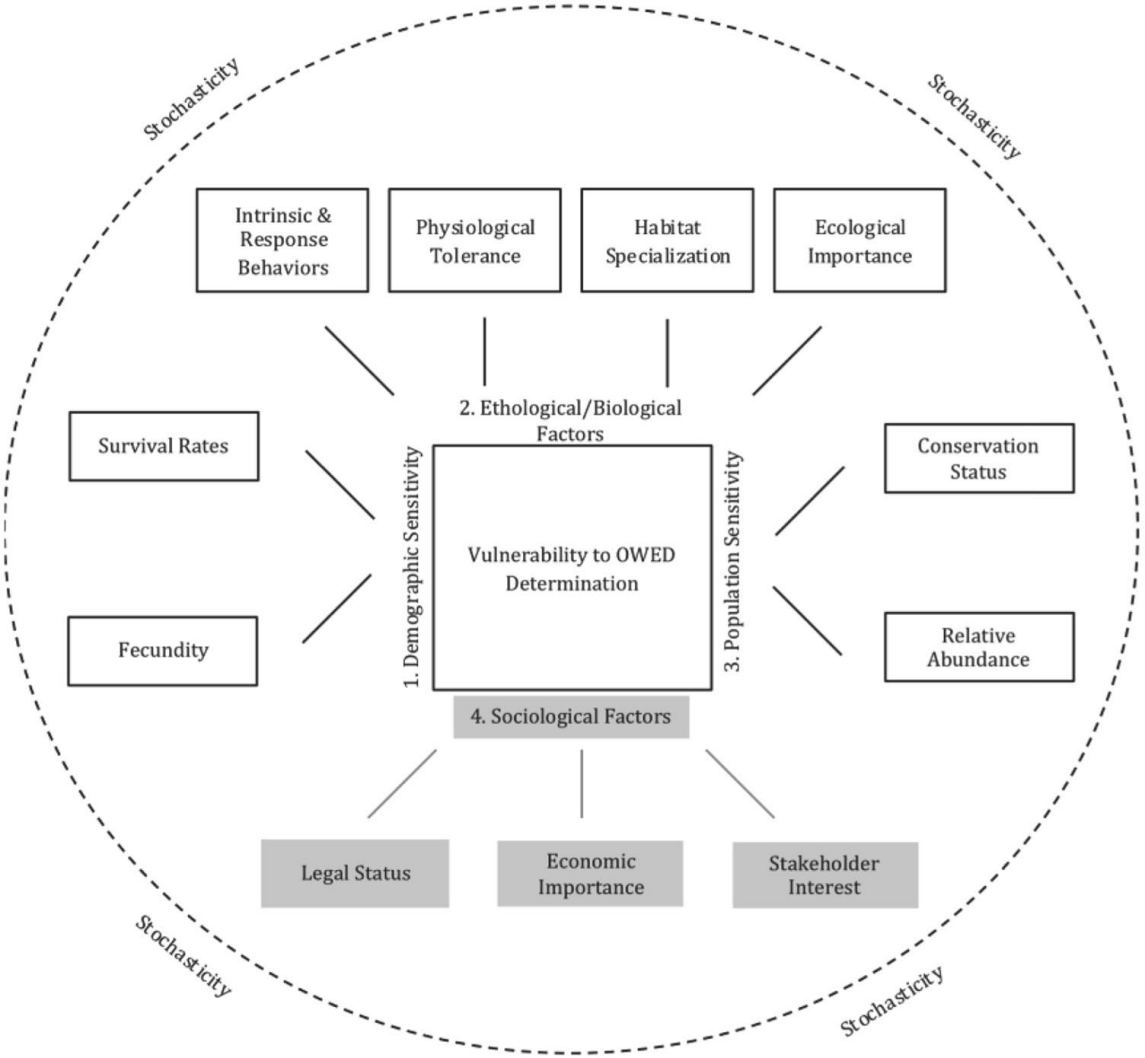
Factors influencing mortality of species by wind farms. The potential negative effects of wind farms depend not only on the characteristics of them (design and spatial distribution of the turbines) but also on the orography of the land, the present species (abundance of the species and also of their prey, their behaviour and their flight characteristics and socioeconomic, demographic aspects (Wing Goodale and Stenhouse^14^).

Other studies indicate that the potential negative effects of wind farms depend not only on the characteristics of them (design and spatial distribution of the turbines)^13^ but also on the orography of the land and the present species (abundance of the species and also of their prey, their behaviour and their flight characteristics^13^. Wing Goodale and Stenhouse^14^ establish, at least, 11 parameters that should be used to assess the incidence on the fauna such as ethological, socioeconomic, demographic and specific aspects of the sensitivity of the species (Fig. 1).

**Figure 2:**
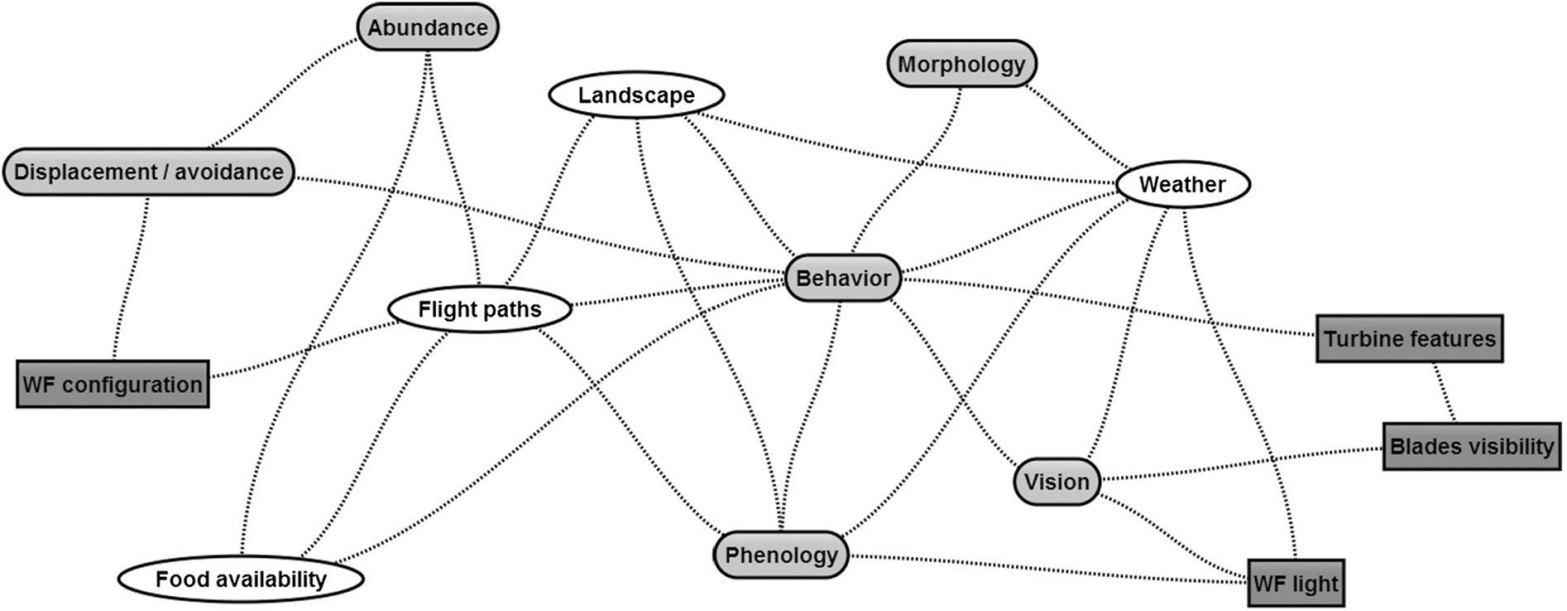
Relationships of variables affecting species mortality. It is necessary to consider the possible relationships between the different variables. Probably, the potential effects of the described wind farms could increase their effects or be mutually exclusive. Reprinted from Marques et al. (2014)^15^, with permission from Elsevier.

Along with all this, we have to consider the possible relationships between the different variables^15^. It is probable that the potential effects of the described wind farms could increase their effects or be mutually exclusive. For example, the risk of collision is greatly reduced if birds alter their habit of using a certain space by avoiding it due to the presence of wind turbines. This means that there are significant differences in flight rates and birdlife risk between the different wind farms and between each of the control points located along each of the wind farms^5,8,16^. In some studies, due to the presence of wind turbines, birds have reacted in risk situations mostly by avoiding to pass and with abrupt changes in their flight paths, affecting in some occasions to individuals mainly migrant^11^ and others in residents^12^ (Fig. 2).

The agreed potential affections are mainly of three types regardless of the variables considered when establishing the incidence of wind farms on fauna^17^:

- Direct loss of habitat as a consequence of the installation of the permanent elements of the wind farm, mainly by wind turbines, evacuation lines, and access and communication roads between turbines.
- Alteration in the behaviour and use of bird area due to disturbances caused during construction and subsequent operation of the wind farm.
- Increased vulnerability to collision with the blades or collision and electrocution with the electrical lines associated with the distribution of the electricity generated.

- Habitat loss
  The direct and permanent loss of habitat due to the construction of a wind farm is evident. However, the adequate selection of land outside the areas designated as sensitive or of interest to birds or bats, minimizes this impact^18^.
- Disturbance and altered behaviour
  Wind farms cause disturbance to wild populations and alterations in their behaviour due to the increased human activity during construction and maintenance. Greater accessibility to the areas where wind farms are located, usually isolated before the installation of the infrastructure, should also be considered. The very presence and operation of wind turbines and the noise of aerogenerators can also deter birds from frequenting certain areas. The alteration of bird habits due to a reduction in the possibilities of using space due to the presence of wind farms is one of the least studied effects, depending to a large extent on the species affected.
  Finally, the location of wind farms can lead to the modification of migratory routes of certain species due to the barrier effect of wind turbine alignments, which must be understood as an alteration in behaviour.
- Collisions
  The direct mortality of birds by the presence of wind farms is due to collisions with shovels, wind turbine towers and evacuation power lines^19^. Occasionally, individuals may fall to the ground because of turbulence generated by blade movement^20,21^. The probability of collision depends on many factors: wind speed, rotor speed and turning radius, type of blades, bird or bat behaviour in front of the wind turbine, etc.^19^, which makes it difficult to calculate it in a generalist context such as the one proposed in this work. In any case, there are several factors inherent to the characteristics of the wind turbines that seem to be more relevant: area of a possible collision, the rotation speed of the turbine blades and extension and location of the wind farm, so these factors will be considered.
  In any case and in relation to the possible collisions of birds with wind turbines, it is worth commenting a series of aspects in a general way:
  - Most accidents occur in poor visibility conditions, at night, at dawn, in areas of bird concentration, and at dusk or on foggy days.
  - The situation of wind turbines is also a factor to consider, being closer or further away from nests, home ranges, rocky areas with birds of prey, etc.
  - In migratory species, there are important factors such as visibility conditions, wind, the migratory route of birds and their level of fatigue.
  These variables cause that the mortality of birds and bats by collision with wind farms varies greatly from one facility to another. For example, in 23 wind farms studied in the USA, the number of raptor deaths per turbine and per year ranges from none to 0.17 ind/turbine^13^.

On the other hand, not all species are equally affected regardless of the wind farm's characteristics or wind turbines. Thus, of the total number of birds surveyed in studies carried out in Navarra in 13 wind farms over 3 years, only 19.4% were birds of prey although they represented 73% of the dead birds, and 63.1% of these were griffon vultures (*Gyps fulvus*)^22^. Also, the golden eagle (*Aquila chrysaetos*) is a species particularly sensitive to the presence of wind turbines based on the studies made by Thelander and Smallwood^23^ in the USA.

However, collision affects not only to large birds due to their flight gliding habits but also some species with gregarious behaviour during their life cycle. For example, in monitoring completed in Navarra, the mortality of the lesser kestrel (*Falco naumanni*) occurred in wind farms close to postnuptial roosts of the species^22^. Barrios and Rodríguez^12^ establish that griffon vultures and common kestrels are the most sensitive species in wind farms located in Tarifa et al.^24^ prove their affection for steppe species in several wind farms in the USA.

We must also bear in mind that the effects on the different species cannot be considered to be the same for demographic, ecological or legislative purposes. For example, the effect on a protected species cannot be considered the same as that on a non-threatened species^25^.

In the end and as it has been possible to verify, there are many considerations that must be taken into account when assessing the impact of wind turbines on flying fauna (birds and bats) and much more if we want to quantify this condition through a mathematical model.

The aim of this work is to establish a mathematical algorithm that will allow us to combine all these variables to quantify the effect that the installation of a wind farm can cause in a given place with specific characteristics. For this purpose we must establish the following objectives: (1) Standardise the specific assessment parameters of wind farms, (2) Determine the most relevant environmental characteristics related to the location of the wind farm, (3) Prioritise morphological, ethological and legal characteristics in the assessment of species and (4) combine the above variables weighing their relevance to establish the final mathematical model.

## Material and methods

Multiple statistical methods have been developed to estimate the effect on birds and bats as a result of wind energy during the last 20 years^26–31^. Some of these studies are focused on the conservation status of the species^32^, the incidence factor of the wind turbines^19,33^, demographic parameters^34,35^, behavioural^12^ and also morphological parameters of the species^36,37^. In any case, it is essential to group all types of affections in order to be able to establish a global quantification that can be adapted to each species and to each specific wind farm. In other words, it can be obtained from a mathematical algorithm that allows quantifying the effect on each species, taking into account the characteristics of each wind farm^3^.

Furthermore, the formula that reflects the effect on the species must consider aspects related to the wind farm itself (type and distribution of turbines, occupation of the territory, etc.) and those related to the species, both in terms of its degree of threat and social importance, as well as its special sensitivity to the presence of the wind farm. According to this, the affection to each species must respond to the following formula:

$${\text{AS}}_{{\text{I}}} = {\text{WF}}\left( {{\text{SS}}_{{\text{I}}} + {\text{VS}}_{{\text{I}}} } \right)$$

where AS_I_ = Affection to species i, WF = Constant derived from the characteristics of the wind farm, SS_I_ = Sensitivity of the species i to the presence of the wind farm, VS_I_ = Social value of species i.

The index of affection, therefore, will be the product of multiplying the obtained values by the wind farm with those of each species that is present in the area.

### Wind farm value constant (WF)

The impact value of the wind farm will be determined by the characteristics of the wind farm and also be influenced by both the characteristics of the wind farm (VWF) and its location (UF). At the same time, the VWF will be determined both by the affection of each wind turbine (WT) and by the distribution within the wind farm (extension and lines of turbines).

$${\text{WF}} = {\text{VWF}} + {\text{UF}}$$

#### Value of the wind farm (VWF)

To calculate the overall effect of the wind farm not only is necessary to know the effect of each turbine but also its distribution in space. It is relevant to assess the distances between the wind turbine and if they are or not operating because when the turbines are very close together, the risk of moving between them is greater than in wind farms with more separate wind turbines^38^ and to know the number of rows in which the turbine are distributed. Crossing a wind farm with a single line of wind turbines is easier than those wind farms with several consecutive rows^39^. For this reason, the global affection of the wind farm (VWF) is understood as:

1. The individual value of each of the wind turbine (WT) multiplied by the number of existing turbines.
2. The total area occupied by the wind farm (AWF); in this way, it is not only considered the whole area of affection but also is established the density of the wind turbines.
3. The number of rows that are included in the wind farm.

Based on the preceding information, the proposed formula for assessing the characteristics of the wind farm is:

$${\text{VWF}} = \left( {\left( {{\text{Ni}}*{\text{WTi}}} \right)/{\text{AWF}}} \right)^{{{1}/{\text{F}}}}$$

where Ni: Number of wind turbines. WTi: Incidence of each wind turbines (the WT value will be the same for all unless in the same wind farm there were different types of wind turbines with different affection areas). AWF: Total surface of the area of study understood as the area formed by the vertical rectangle created between the furthest wind turbines from the same front line and the height of them. In the case of wind farms with more than one row, the total surface area is calculated as the sum of the surfaces of each row. F = Number of lines forming the wind farm.

Of these variables considered in the previous formula, it is only necessary to develop the affection inherent to each wind turbine (WT) that has to be calculated considering both the area of the turbine’s affection and the rotation speed of the blades.

$${\text{WT}} = {\text{AFM}}*{\text{BRS}}$$

where AFM: Area of affection of each wind turbines, BRS: Blade rotation speed.

**Figure 3:**
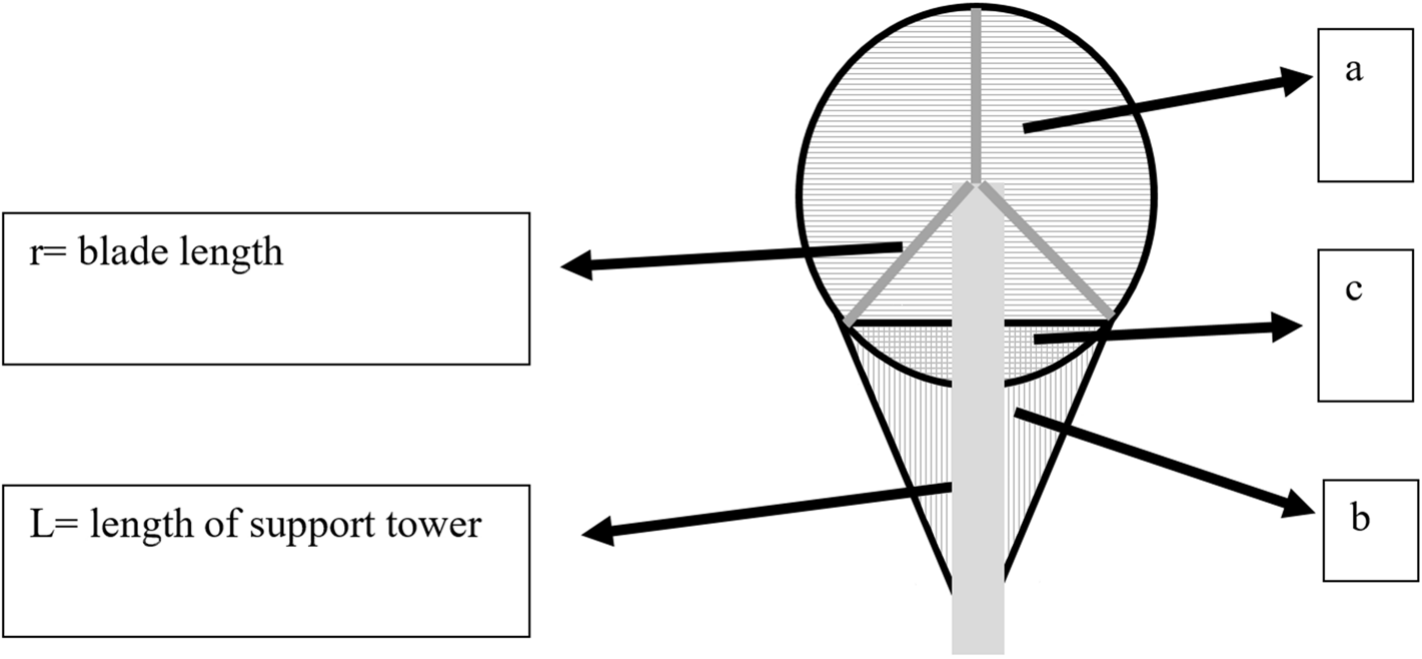
Scheme and values to calculate the area of affection of each wind turbine. The area of affection of each turbine is the surface of the circumference formed by the blades (a), plus the surface of the triangle formed by the blades with the ground when they form an angle of 60° with the support tower (b), minus the intersection of both surfaces (c).

The area of affection of each turbine is the surface of the circumference formed by the blades (a), plus the surface of the triangle formed by the blades with the ground when they form an angle of 60° with the support tower (b), minus the intersection of both surfaces (c) (Fig. 3):

$${\text{AFM}} = {\text{a}} + {\text{b}}{-}{\text{c}}$$

**Figure 4:**
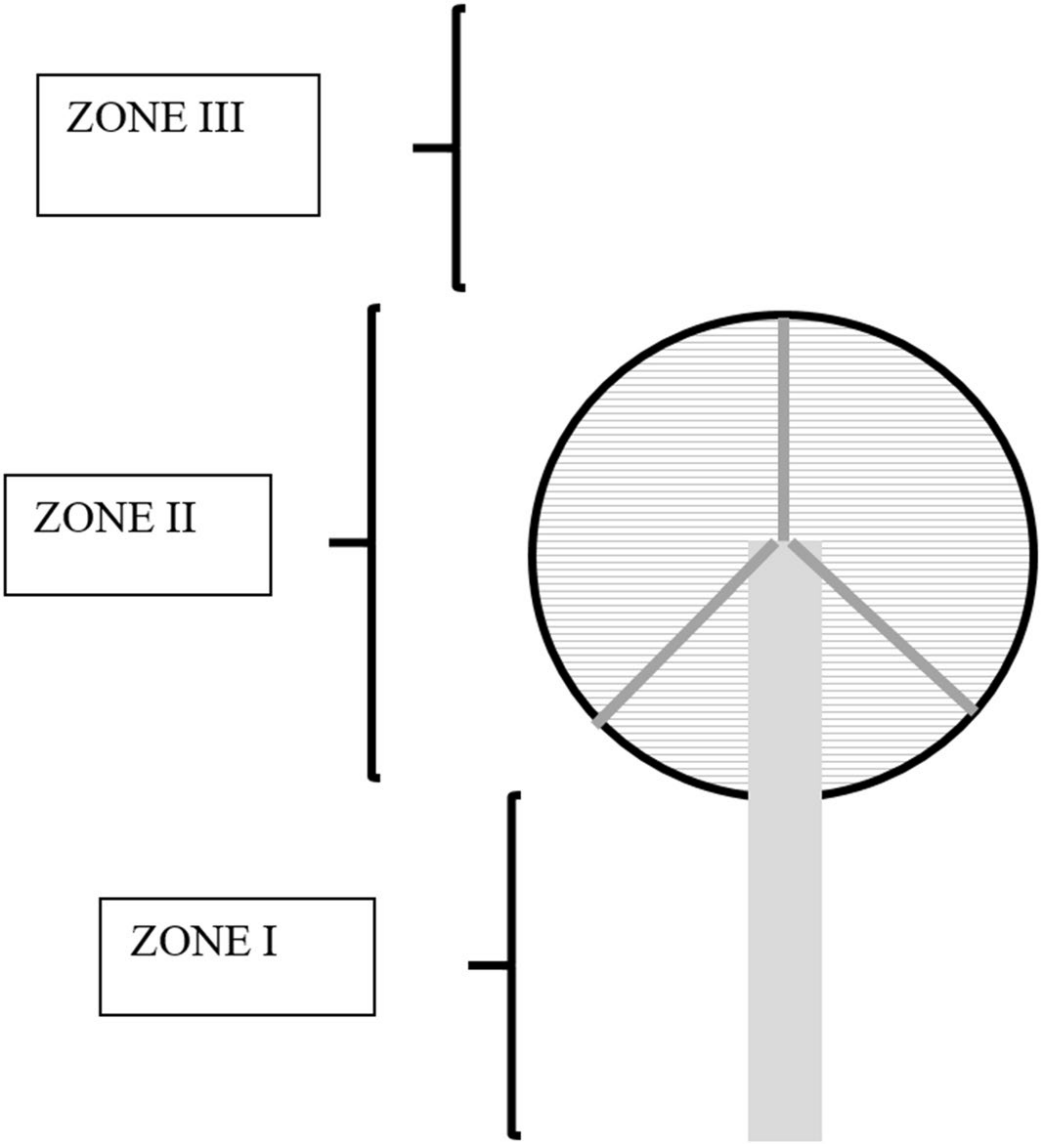
Zoning scheme of risk areas. ZONE I: Corresponds to the free height between the ground and the blades. ZONE II: This zone corresponds to the area of the circumference formed by the blades when turning. ZONE III: Corresponds to the free height above the blades so that this interval is above the previous interval.

Being: a = πr^2^   b = (sen60°*r)(L − cos 60° * r), where r is the length of the blades and L the height of the support tower. c = ((πr^2^/3) − ((sen60° * r)(cos 60° * r))).

To calculate the affection of the rotation speed of the blades (SB) it is assumed that the greater the rotation speed, the greater the turbulence and the greater the risk for the fauna^19,40^. In any case, this incidence is not linear but exponential since from a certain speed the affection can be considered high. To calculate this value, we established the following formula:

$${\text{BRS}} = {1} + {\text{Log}}\left( {{\text{SB}}} \right)$$

Given that the value of the wind Farm (VWF) is the quotient between the sum of the areas affected by each turbine and the total area occupied, the value generally will be less than 1. In cases where the value is greater (when the surface area of the turbine multiplied by the rotation speed of the turbine is greater than the total surface of the area) it will be equal to 1. i.e., the maximum surface area affected cannot be greater than the surface area occupied by the total wind farm.

#### Location in the natural environment (UF)

Many works show the importance of selecting the location of the wind farm to minimize its impact on birdlife. However, it is possible that wind farms may be authorized in sensitive areas or in areas with poor environmental conditions (predominance of fog) or that have synergistic effects with adjacent wind farms. In this sense and as indicated in the introduction, there are four factors that can influence this impact: low visibility, proximity to sensitive areas, location in migratory crossings and synergies with other wind farms. Therefore, the value of this variable should be at least the same as that established for the previous variable (VWF). In this regard, it is proposed that the maximum value of the variables used to compute this factor should also be 1.

- Visibility (VI): This variable measures the frequency of days with low visibility (fog, intense rain, etc.) compared with the total number of observation days (total number of days with low visibility/total number of observation days). The maximum value is 0.25.
- Proximity to sensitive areas (ZS): Sensitive areas are those in which occur high concentrations of individuals, either because they are breeding areas, feeding areas, resting areas or roosts. Protected areas such as IBAS or LICs may also be considered. Not all species have the same radius of action, so setting a minimum radius of affection can only be established randomly. For example, for some species a radius of influence of 10 km is small but for others can be large. In any case and for having a uniform criterion, it will be considered that a sensitive area is close to the wind farm when it is located less than 10 km^4^, in this case, the value of this variable will be 0.25 and if they are between 10 and 50 km the value will be 0.15 while if it is more than 50 km is considered that the location of the wind farm does not influence these areas (value 0).
- Migratory passes (MP): Migratory passes are those areas used by avian fauna for their daily or migratory movements. If the wind farm is located in one of these Migratory passes, the effect will be high so it will be valued with a maximum value (0.25) and the value will be minimal (0) if this is not the case.
- Proximity to other wind farms (PWF): It is relevant to include this variable because of the proximity of different wind farms cause negative synergistic effects on the species by limiting the length of possible free corridors of wind turbines. In this way, the location of another wind farm less than 3 km away is considered very negative (0.25), between 3 and 5 km (0.15), between 5 and 10 km (0.10) and more than 10 km (0), it does not affect. If there is more than one wind farm in the area, the value will increase 0.25 if it is between 3 and 5 km and 0.15 if it is between 5 and 10 km.
  $${\text{UF}} = {\text{VI}} + {\text{ZS}} + {\text{MP}} + {\text{PWF}}$$
  where WT: Value related to the location of the wind farm. VI: Predominant visibility in the area. ZS: Presence of sensitive areas in the vicinity of the wind farm. MS = Incidence of the wind farm in migratory crossings. PWF: Proximity to wind farms.

The possible maximum value for the wind farm location will be 1.

Therefore, the possible maximum value inherent in the characteristics and location of the wind farm will be 2. Substituting the values in the proposed formula:

$${\text{WF}} = {\text{VWF}} + {\text{UF}}$$

And considering the values obtained for each mill, the wind farm in general and its location, the result is the following formula:

$${\text{WF}} = \left( {\left( {{\text{Ni}}*{\text{IMi}}} \right)/{\text{AWF}}} \right)^{{{1}/{\text{F}}}} + \left( {{\text{VI}} + {\text{ZS}} + {\text{MP}} + {\text{PWF}}} \right)$$

### Affection on the species

Not all species have the same sensitivity to the presence of the wind farm, being some of them more sensitive than others (25). On the other hand, the incidence on endangered species is not the same as that on species with stable populations in the area so, it is necessary to differentiate two types of variables related to the species: those related to the special sensitivity of each species to the presence of these infrastructures (SS) and the one inherent to its degree of threat, conservation or socioeconomic interest (VS). The affection value of each species will be the sum of the values of each type of variable. Therefore, the value of this section will be:

$${\text{Affection}}\;{\text{to}}\;{\text{the}}\;{\text{species}} = \left( {{\text{SS }} + {\text{ VS}}} \right)$$

#### Sensitivity of the species to the wind farm (SS)

These variables will be considered as the impact of the wind farm on each species due to its morphological, ethological, historical and demographic characteristics, etc. It is the closest thing to what could be understood as collision risk since it assesses the different characteristics of each bird (morphological, ethological, demographic, etc.) based on the risk of colliding with wind turbines and, valuing more those characteristics that enhance the probability of collision.

- Morphological variables (Maximum value 1).

Bird size will be considered in this variable. A higher percentage of affection is detected on large birds in the majority of the recorder monitoring of the incidence of wind farms. However, this value seems to be overestimated since the detectability of carcasses of small birds and bats is lower as they remain less time on the ground^30,41,42^.

On the other hand, small birds show much less resistance to wind flows generated by the blades so it seems logical to think that the affection on this group of birds and on bats is higher. For this reason, a greater impact on small species has been assessed.

As a reference size, those birds smaller than or equal to a turtledove have been considered as small birds; medium-sized birds are those whose sizes are between a turtledove and a heron while those larger than a heron are considered as large birds. Considering these aspects:

- Bird size (small 1; medium 0.75; large 0.5).

- Ethological variables (maximum value 4.5).

The behaviour of different species will influence their risk of collision increasing the possibility of being affected by wind turbines^38^, for example, species that tend to go in groups show a greater risk of collision. The phenological characteristics of species are also important, for example, those species that are only in passage (prenuptial and postnuptial) will be little time in the study area but as they are not accustomed to the presence of wind turbines, probability of collision is high and possibly increased by going in large groups. Breeding species in the area are more dangerous because the young ones, still inexperienced in flight, show high risks of collision^1^. In other words, variables reflected in this section are related to the time the species spends in the area^38^, its dexterity in flight and its gregariousness. Together with these variables, the type of flight carried out by each species has been also considered: direct flights avoid staying longer in the area while cycloid or indirect movements increase the possibility of collision.

- Seasonality: It considers the number of months in which the species is detected in the area. The maximum value is 1 if the species is sedentary (12 months) so each month is valued as 0.083.
- Phenology: Marks the periods in which the species is present in the area. It is considered that species present in the breeding season or in passage show a greater risk than those that are only wintering. In this sense, if the species is in the breeding season will be valued with 0.75, only in winter 0.25 and 0.5 only in passage. When it appears in all seasons or in three of them, the value will be maximum (1). The value of the station will be also maximum if appears in two periods.
- Flight height: In order to calculate flight height with risk for each species, the characteristics of each wind farm are considered. That is to say, they have to be adjusted to each wind farm since the interval of each zone will vary according to these ones. In this sense, for example, small birds that fly at lower altitudes can be located in the lower zone or not depending on the wind farm, just as large birds can be located in the area of the blades or above. In this sense, three zones have been established (Fig. 4):
  - ZONE I: Corresponds to the free height between the ground and the blades so, this interval goes from 0 m to the height resulting from subtracting the size of the blade from the length of the support tower. Value 0.5
  - ZONE II: This zone corresponds to the area with the greatest risk of collision since it is equivalent to the circumference formed by the blades when turning. Therefore, the interval will go from the previous height to its sum with the diameter of the circumference formed by the blades. Value: 1.
  - ZONE III: Corresponds to the free height above the blades so that this interval is above the previous interval. Value: 0.

When a species presents different flight heights, the one more frequent and that presents the greater risk will be selected.

- Type of flight: Direct flights are considered to have a lower risk of collision than those that cause a longer stay in the area. The values will be 0.25 in direct flights and 0.5 in indirect flight.
- Flock size: The risk of collision is considered higher when species show large groups so the following classification is established: One individual: 0.25; groups of 2–5 individuals: 0.5; groups of 6–10 individuals: 0.75; groups of more than 10 individuals: 1.
- Historical variables (Maximum value 2).

A variable related to mortality detected in previous studies has also been included. Those species that are systematically detected in the mortality reviews of these infrastructures or exist high figures of mortality due to collision in specific wind farms should be considered.

- Species with previous collision data (usual 2; medium 1; scarce 0.5; no record 0). This value is established at the discretion of the technician who performs the assessment, but as a habitual criterion, it can be considered as usual when the species appears in most of the studies (more than 30% of the studies), between 15 and 30% of the studies on average; and it will be classified as scarce if it only appears between 1 and 15% of the studies.

- Demographic variables (Maximum value 2.5).

The last variables considered are related to the incidence on population parameters of each species. It has been considered that the species with reproductive strategy R suffer a lower incidence on the populations (although the mortality may be higher) since their reproductive efficiency partly solves this loss. However, species with K strategy suffer enormously when the mortality of young individuals is high. On the other hand, those species that frequently use the area where the wind turbines are located will show a higher probability of collision than those that are less common and those species that have high abundances in the area have also a higher probability of impact than those with few individuals^16^.

- Survival-Fertility (type K or R) (K = 0.5; R = 0.2).
- Frequency: This variable measures the frequency with which each species appears in the area in relation to the rest of the species present (total number of presences of the species/total number of presences detected). The maximum value is 1.
- Abundance of the species in the area (number of individuals detected of the species i/total number of individuals detected of all species) (maximum value 1).

#### Species value (VS)

This value will include the conservation and socio-economic importance of the species (including the hunting value or social interest of some species). The affections on those species that are in a situation of greater risk of extinction must be considered in a relevant way, since the loss of a few individuals can represent the unfeasibility of the population. In this respect, both the degree of threat and the legal cataloguing of the different species have been considered.

The maximum value of this variable is much higher than the rest of variables since those species with the maximum protection value or degree of threat will have a value of 9. The cataloguing according to the Red Books will relate to the value established in Table 1^43^. It has also been considered necessary to assess the socio-economic importance of some species. In this regard, it is taken into account not only the importance of hunting, which is relevant for some species of birds, but also its social importance, that is to say, those species which have conservation or recovery plans established in areas close to the different administrations or which are especially valued by the population, although their threat level is not very high (colonies of birds especially loved by the local population, etc.).

- Variables of conservation importance (Maximum value 9).
  - Red book.
- Socio-economic variables (Maximum value 1).
  - Species with social value (Species with management or economic interest = 1; Species without management or economic interest = 0). In this category will be included hunt species or species with conservation or recovery plans or even with cultural interest.

**Table 1:** Values given to the different classifications or threat level. (Classification modified from Refoyo^43^).

Degree threat (Red Book)	Value
CR: Critical hazard	9
E: Endangered	8
V: Vulnerable	6
NT: Near threatened	4
DD: No data	2
LC: Not threatened	1
NE: Not evaluated	1
NC: Not catalogued	1

## Results

According to the established formula, each section presents an independent assessment. Thus, the WF Constant can reach a maximum value of 2 (1 for VWF and 1 for UF) which means that the most dangerous wind farms will multiply by 2 the related value for each species. Likewise, the value of each species will be determined by the sum of its sensitivity values SS (whose maximum value is 10) and its inherent value to its conservation status or interest VS (whose maximum value is also 10).

Two types of valuations are established for the definitive valuation. Total Valuation and Weighted Valuation.

*Total Valuation* According to the established methodology, the resulting valuation scale is between 1 and 40. Obtaining a value of 1 is practically impossible. The maximum possible value for a species would be that obtained by multiplying the value of the wind farm (maximum 2), and the one of the species (maximum value 20), i.e. 40, which is also very unlikely to be obtained. For this reason, a Total Valuation is established on the basis of a reference scale that will allow us to establish categories of affection for the different species. Given the almost impossibility of obtaining the maximum standardised value (40), an increasing classification has been considered by adding the increasing value to the maximum value of the previous category, as shown in Table 2.

**Table 2:** Classification of birds according to their sensitivity to the wind farm.

Class	Obtained value	Increasing value
Class I: low sensitivity	1–5	
Class II: sensitive	5.1–10	5
Class III: very sensitive	10.1–20	10
Class IV: extremely sensitive	20.1–40	20

Obtaining minimum and maximum value is practically impossible, so a Total Valuation is established on the basis of a reference scale that will allow us to establish categories of affection for the different species. An increasing classification has been considered by adding the increasing value to the maximum value of the previous category.

*Weighted Valuation* We also propose the establishment of a weighted valuation of the results. With this type of valuation, we will be able to establish the hierarchy of effect on the different species present in the area. The species that shows the highest value will be given the value 1 and the rest of species will have a weighted value with respect to this maximum value.

## Conclusions

Nowadays, wildlife researchers have a relatively good understanding of the causes of bird collisions with WT, however, it is still difficult to make accurate predictions of risks of collision^15^.

Due to the complexity of the factors influencing this collision risk, mitigating bird mortality is not an easy task^15^. There are numerous approaches to avoid, minimize or mitigate impacts through compensatory measures available to wind energy developers^44^. On the other hand, there are substantial models that attempt to predict collision risk^41^ although most of them are based on subsequent data to the location of wind farms since prior data are often not available, and it is unlikely that they will be available in the near future for most wild species.

There are also models that facilitate the gradual incorporation of information, so ecologists, managers, policymakers and industry that are not familiar with a Bayesian analytical to assessing and draw inferences from the model outputs can use them, however, they are often applied to specific species^45^.

Therefore, wind operators and regulators should apply the precautionary principle^44^, and use all possible tools to predict both the impact on species and their sensitivity to the location of wind farms.

An effective way to mitigate this impact would be to establish universal formulas to predict impacts when planning a new facility and when assessing the environmental impact of a future project.

However, there are currently no such universal formulas for predicting impacts, let alone a mathematical model for establishing the sensitivity of different species to the presence of a specific wind farm located in a determined space, something that should be a priority when planning the location of different wind farms and so, selecting the least harmful alternative for fauna.

This work combines biological factors (demographic, ethological/biological and population sensitivity factors) with sociological factors and characteristics of the projected wind farm to determine its impact on avian fauna, considering the specific sensitivity of each species.

The use of the proposed mathematical formula will allow operators, wind farm regulators and stakeholders to know the possible effect of the installation on the fauna species present in the area beforehand and take the most appropriate decisions to minimize the risks to biodiversity.

This new formula can be effective in the determination of birds and bats sensitivity to wind farms as it considers a large number of variables (having also into account in numerous papers) and can be applicable to all affected species.

We are aware of the difficulty of quantifying all the variables and their interactions in this type of generalist models, however, we hope that the scientific community can improve and apply it in the evaluation of future wind farms, as well as in those already installed.
