# Author Correction: Assessing the effect of wind farms in fauna with a mathematical model

**DOI:** 10.1038/s41598-021-93140-9

**Published:** 2021-06-25

**Authors:** Pablo Refoyo Román, Cristina Olmedo Salinas, Benito Muñoz Araújo

**Affiliations:** Biodiversity, Ecology & Evolution Department, Biological Sciences Faculty, Complutense University of Madrid, José Antonio Novais, 12, 28040 Madrid, Spain

Correction to: *Scientific Reports* 10.1038/s41598-020-71758-5, published online 08 September 2020

The original version of this Article contained an error in Figure 2’s legend, where the copyright for the image was not acknowledged.

Consequently, Figure 2’s legend,

“Relationships of variables affecting species mortality. It is necessary to consider the possible relationships between the different variables. Probably, the potential effects of the described wind farms could increase their effects or be mutually exclusive (Marques et al.^15^).”

now reads:

“Relationships of variables affecting species mortality. It is necessary to consider the possible relationships between the different variables. Probably, the potential effects of the described wind farms could increase their effects or be mutually exclusive. Reprinted from Marques et al. (2014)^15^, with permission from Elsevier.”

The original Article has been corrected.

